# Author Correction: Land-use change interacts with climate to determine elevational species redistribution

**DOI:** 10.1038/s41467-020-17319-w

**Published:** 2020-07-08

**Authors:** Fengyi Guo, Jonathan Lenoir, Timothy C. Bonebrake

**Affiliations:** 1 School of Biological Sciences, The University of Hong Kong, Pokfulam, Hong Kong SAR, 999077 China; 2 UR “Ecologie et Dynamique des Systèmes Anthropisés” (EDYSAN, UMR 7058 CNRS-UPJV), Université de Picardie Jules Verne, 1 Rue des Louvels, 80037 Amiens Cedex 1, France

**Keywords:** Biogeography, Climate-change ecology, Conservation biology

Correction to: *Nature Communications* 10.1038/s41467-018-03786-9, published online 3 April 2018.

Since publishing the original Article, we became aware that we made a mistake in transcribing data from Dainese et al.^1^ into Supplementary Data 2. Specifically, we mistakenly logged the original shift rates (m/yr) from Dainese et al.^1^ as the total shift extent (m), affecting the disaggregated analyses using this dataset at the species level. When we re-ran these analyses using the corrected dataset, we obtained poor model fits, likely due to the unusually high shift rates and large variance driven by human-assisted dispersal in Dainese et al.^1^, which dominated the dataset. Therefore, we decided to exclude the Dainese et al.^1^ data points in the disaggregated, species-level component of our analyses. Although this error alters the species-level results, it does not affect the aggregated, site-level results and the main conclusions of this study.

The text below details the changes that have been made to the relevant parts of the paper involving the disaggregated analyses, by section.

Corrections to the sample size throughout the paper

The incorrect sample size of *n* = 2798 has been replaced with *n* = 1464 in the third sentence of the “Abstract,” the last paragraph of the “Introduction,” the “Disaggregated analysis” subsection of the “Results,” and the legend for Fig. 4.

Corrections in the “Methods” section

At the end of the second sentence of the first paragraph in the “Disaggregated analysis” subsection, we now add the following new text: “The species-level shift dataset is dominated by records from a single study site (Dainese et al.^1^, *n* = 1334 out of 2798), of which the rapid shift rates were associated with proximity to roads and the occurrence of several non-native species. The goodness-of-fit of models including these data points are low ($R_{\mathrm{conditional}}^{2}$ < 0.02, $R_{\mathrm{marginal}}^{2}$ < 0.05). Therefore, we excluded data from Dainese et al.^1^ and only used data from other study sites in the following analyses (*n* = 1464).”

The fifth sentence of the first paragraph incorrectly stated: “As the raw mean shift extent across species was 36 m, …”. This has been replaced with the correct average value: “As the raw mean shift extent across species was 66 m, …”.

The twelfth sentence of the second paragraph in the “Disaggregated analysis” subsection incorrectly stated: “…baseline temperature and forest cover tend to have site-specific impacts on individual species’ shift rates (i.e., the 1 + T +  Cover|Site random structure showed the lowest AICc value).” This sentence has been replaced with the correct random component: “…forest cover tends to have site-specific impacts on individual species’ shift rates (i.e., the 1 + Cover|Site random structure showed the lowest AICc value).”

The first sentence of the “Sensitivity analysis restricted to forest systems only” subsection incorrectly stated: “…we reran the above analyses at both site (*n* = 29) and species (*n* = 2419) levels on forest systems only…”. This has been replaced with the correct sample sizes: “…we reran the above analyses at both site (*n* = 29) and species (*n* = 1120) levels on forest systems only…”.

Corrections in the “Results” section

The main results figure pertaining to this analysis is Fig. 4 from the original Article. The correct version of Fig. 4 is:



which replaces the previous incorrect version:



The correct legend for Fig. 4 is:

“Coefficient averages of the five most important predictors with 95% confidence intervals. Models constructed using the full dataset (n = 1464). For details of the 30 competing models refer to Supplementary Table 3. All variables are scaled to allow direct comparison both in direction and in magnitude, ranked by importance (threshold = 0.6). Sdist: distance to mountain summit, T: baseline temperature, CCR: climate change rate, Cover: forest cover percentage.”

Which replaces the previous incorrect version:

“Coefficient averages of the four most important predictors with 95% confidence intervals. Models constructed using full dataset (*n* = 2798). For details of the 13 competing models refer to Supplementary Table 3. All variables are scaled to allow direct comparison both in direction and in magnitude, ranked by importance (threshold = 0.7). Sdist: distance to mountain summit, T: baseline temperature, Cover: forest cover percentage, Ref: reference point (margin or center).”

In the “Disaggregated analysis” subsection, number of candidate models has been changed from 13 to 30.

The main results of the disaggregated analysis using the updated and corrected species-level dataset are similar to the original ones, but there are some changes affecting the text. The original fourth through sixth sentences of the “Disaggregated analysis” subsection read:

“Our findings at the species level suggest increasing shift rates for species with denser baseline forest cover, for higher baseline temperature conditions, and for greater elevational distance to the highest mountain summit within the study area. Although the data suggest that the magnitude of the elevational shift rate might be higher at the margins than at the core of the distribution, this effect was not significant (see the large 95% confidence interval crossing the zero line for “Ref_Margin” in Fig. 4). Interestingly, when restricting data to forest systems only (forest cover >25%, *n* = 2419), we found significant synergistic effects between baseline temperature and forest cover, as well as between climate change rate and forest loss percentage on elevational shift rate at the species level (Supplementary Fig. 2).”

This incorrect text has now been replaced with:

“Our findings at the species level suggest increasing shift rates for species with higher baseline temperature conditions, and for greater elevational distance to the highest mountain summit within the study area. Although the data suggest that the magnitude of the elevational shift rate might be higher under denser baseline forest cover conditions, this effect was not significant (see the 95% confidence intervals crossing the zero line for “Cover” in Fig. 4). The data also suggest that the magnitude of the elevational shift rate might be affected by synergistic effects between climate change rate and baseline temperature, but these effects were also not significant (see the 95% confidence intervals crossing the zero line for “CCR” and “CCR * T” in Fig. 4). Interestingly, when restricting data to forest systems only (forest cover > 25%, *n* = 1120), we found significant synergistic effects between climate change rate and forest loss percentage on the elevational shift rate at the species level (Supplementary Fig. 2).”

Corrections in the “Discussion” section

Several minor changes have been made throughout the “Discussion” section.

In the first sentence of the first paragraph, the phrase “confounding impacts of habitat features (i.e., forest cover)” has been changed to “confounding impacts of habitat features (e.g., forest cover, forest loss)”.

In the first sentence of the second paragraph, the phrase “the generally positive effects of forest cover” has been changed to “the generally positive effects of forest cover (although not significant at the species level).”

The fourth sentence of the second paragraph, “In addition, when restricting the disaggregated analysis to forest systems only, the synergistic effect between forest cover and temperature further confirmed the importance of habitat connectivity in enhancing species movement16” has been deleted, because there is no longer a synergistic effect between forest cover and temperature after updating the dataset at the species level.

In the third sentence of the fourth paragraph, the phrase “unexplained variance in our models at the species level (cf. 76.4% on average, with ±0.65% SD)” has been changed to “unexplained variance in our models at the species level (cf. 87.4% on average, with ±0.97% SD).”

Corrections in the Supplementary Information file

The original, incorrect, version of the Supplementary Information is attached to this Correction. The new, correct, Supplementary Information file contains updated versions of Supplementary Fig. 2, Supplementary Fig. 7, Supplementary Table 3, and Supplementary Table 5.
