# Supplementary material for: Land-use change interacts with climate to determine elevational species redistribution
Source: Nat Commun. 2018 Apr 3;9:1315. doi: 10.1038/s41467-018-03786-9 (PMC5883048; doi:10.1038/s41467-018-03786-9)
Supplement: Supplementary file 1 — Supplementary Information(PDF 1689 kb) [file 41467_2018_3786_MOESM1_ESM.pdf]

# Land-use change interacts with climate to determine elevational species redistribution

Guo *et al*.

## Supplementary Methods

We obtained global mountain range boundaries (n = 140) from Natural Earth's physical vectors (http://naturalearthdata.com/) and constructed the forest cover (%) profiles of each mountain range following the protocol on mountain hypsographic curves by Elsen & Tingley (2015). Specifically, we overlaid high resolution global forest cover (at year 2000) data from Global Forest Watch (Hansen et al. 2013, http://earthenginepartners.appspot.com/science-2013-global-forest) and climate data from WorldClim (Hijmans et al. 2005, bio1 at 2.5 arc-min which is about 5 km resolution at the equator, http://worldclim.org/version2) on to digital elevation models (SRTM Arc-Second Global, about 30 m resolution at the equator, https://lta.cr.usgs.gov/SRTM1Arc) of each mountain range, and generated forest cover as well as temperature profiles along the elevational gradient (at 100 m elevation band intervals). To assess the autocorrelation signal of the proportion of forest cover or mean annual temperature per elevational band along the entire elevation gradient, we computed a correlogram for each mountain range separately, using the autocorrelation function "acf" in RStudio (R Core Team, 2016). The generalized pattern for global mountain ranges for both forest cover and mean annual temperature and their corresponding autocorrelation signals were obtained by taking the average of all 140 mountain profiles at the corresponding elevation band and elevational distance, respectively. Apart from the mean trend, variation among mountain ranges were also represented by the ±0.5 standard deviation.

## Supplementary Figures

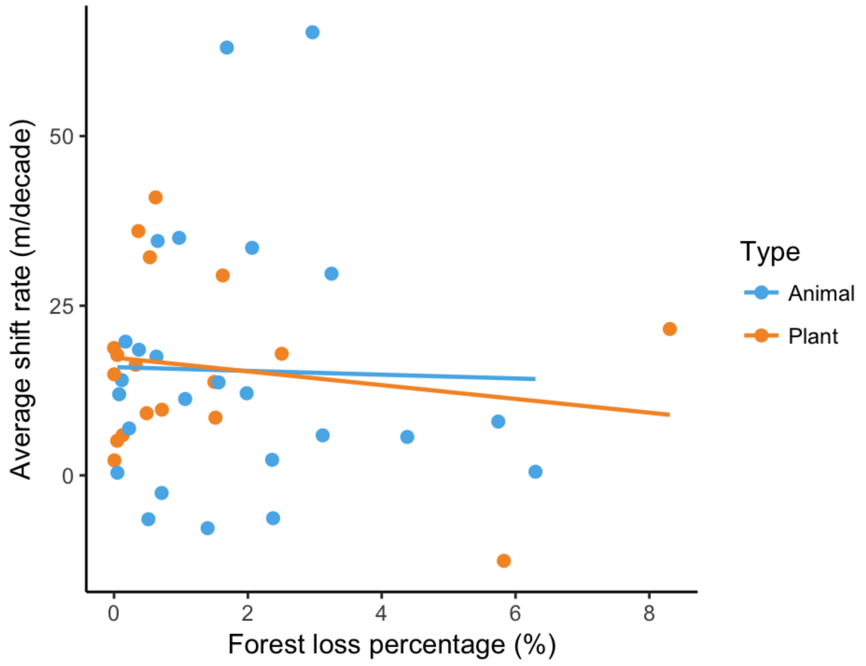


***Supplementary Figure 1. Interaction effect between forest loss and taxa type (Animal or Plant) on species elevational shift rate averaged at the site level (n = 43)*** *Data were plotted in a natural scale for a straightforward display of the relationship between each explanatory variable and the average shift rate. Blue dots and regression line represent animal shift rates while orange represents plants. Note that other covariates in this model (cf. Model 3 in Tables 1 and 2) were set to their mean values.*

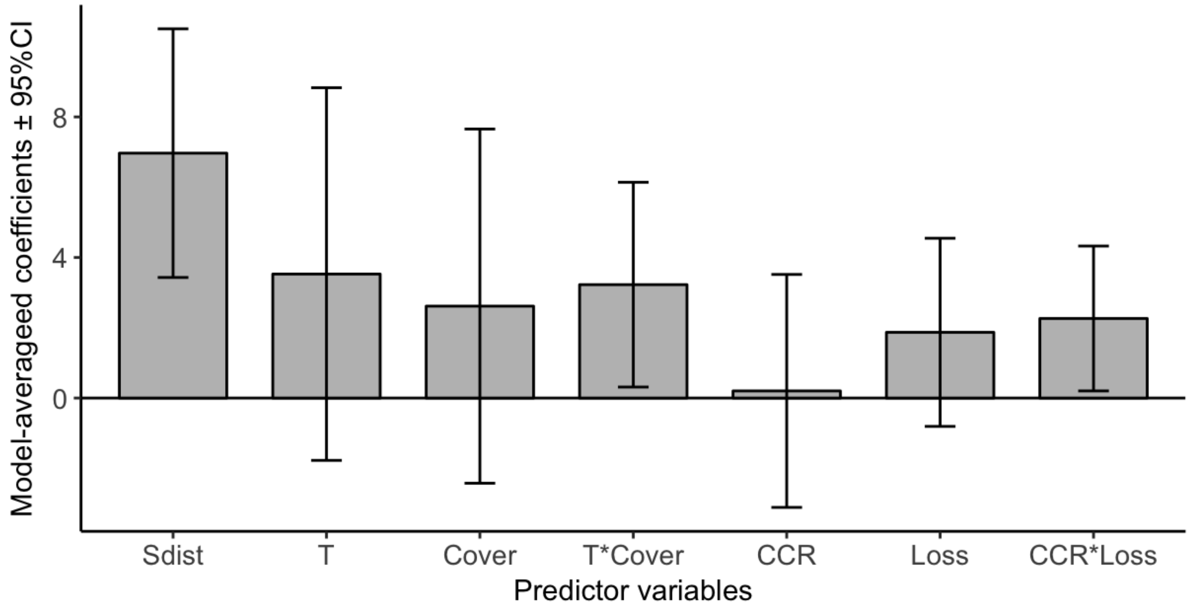


***Supplementary Figure 2. Coefficient averages of the top 7 most important predictors (importance>0.7, data restricted to forest ecosystem only, n = 2419) with 95% confidence intervals.*** *All variables are scaled for direct comparison purposes in both direction and magnitude, ranked by importance. A confidence interval crossing the zero horizontal means that the corresponding focal variable is non-significant.*

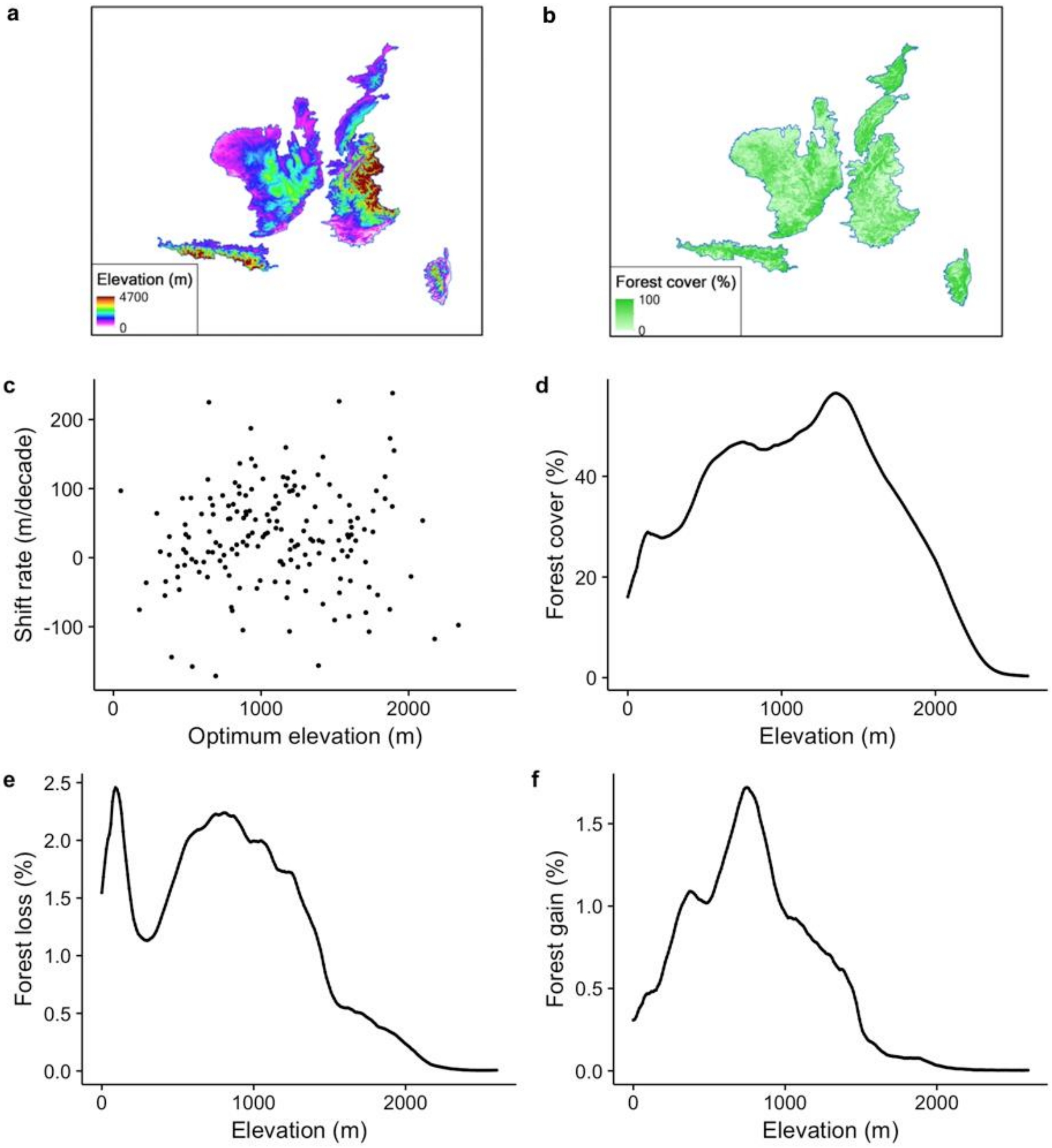


***Supplementary Figure 3. Mountain profiles based on the study case of Lenoir et al. (2008).*** *Elevation (a) and forest features (b) are displayed inside the digitized polygons corresponding to the study area extracted from Lenoir et al. (2008). Scatterplots show: the overall elevational shift rate of each studied species (n = 171) across the studied area at its historical optimum elevation location (c); forest cover by 10-m elevational band (d); and forest cover change by 10-m elevational band (e and f).*

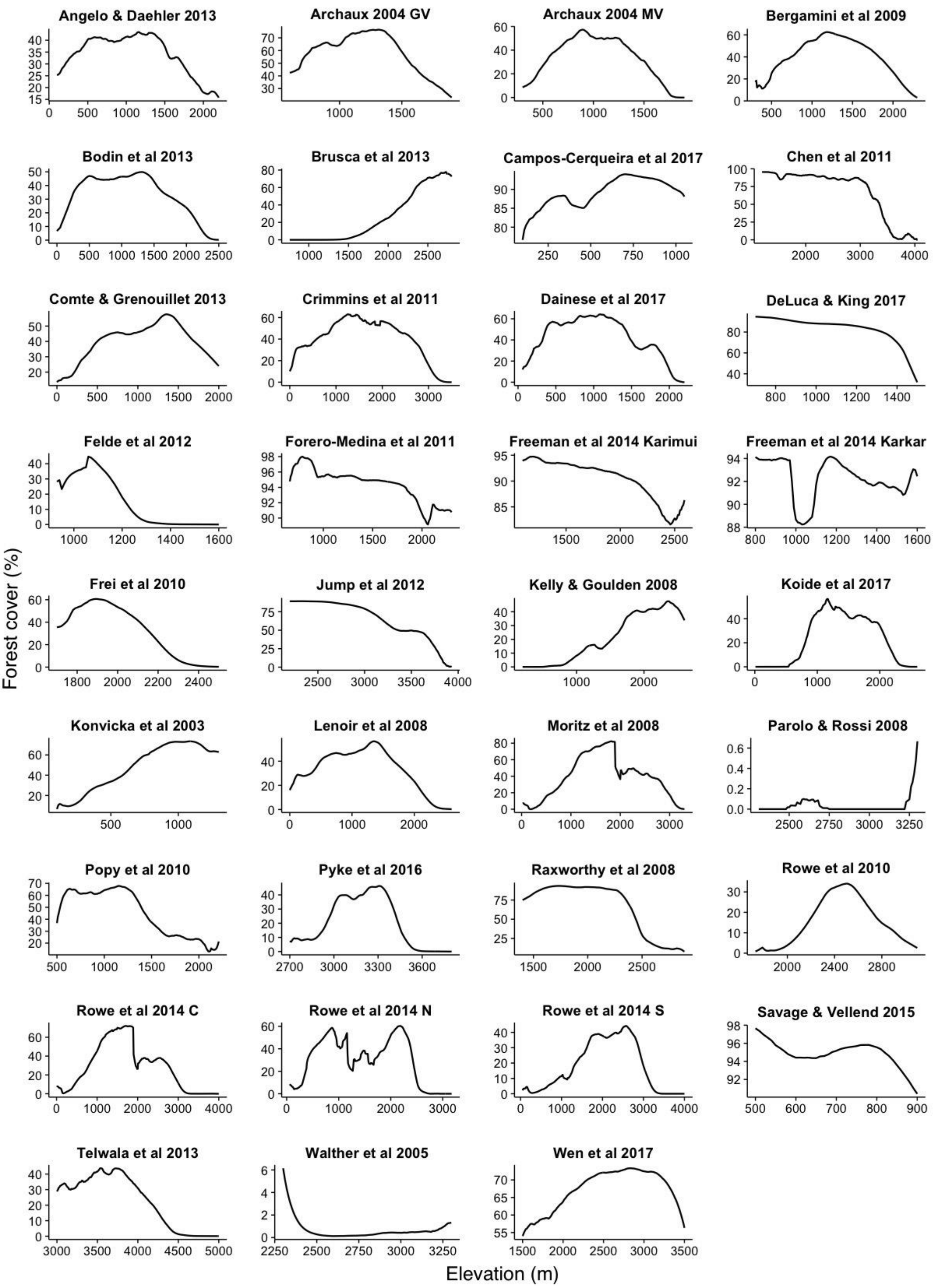


***Supplementary Figure 4. Forest cover (%) profiles across elevation (10-m band) for the 35 study sites with species-level shift data.*** *Data plotted within the studied elevation range.*

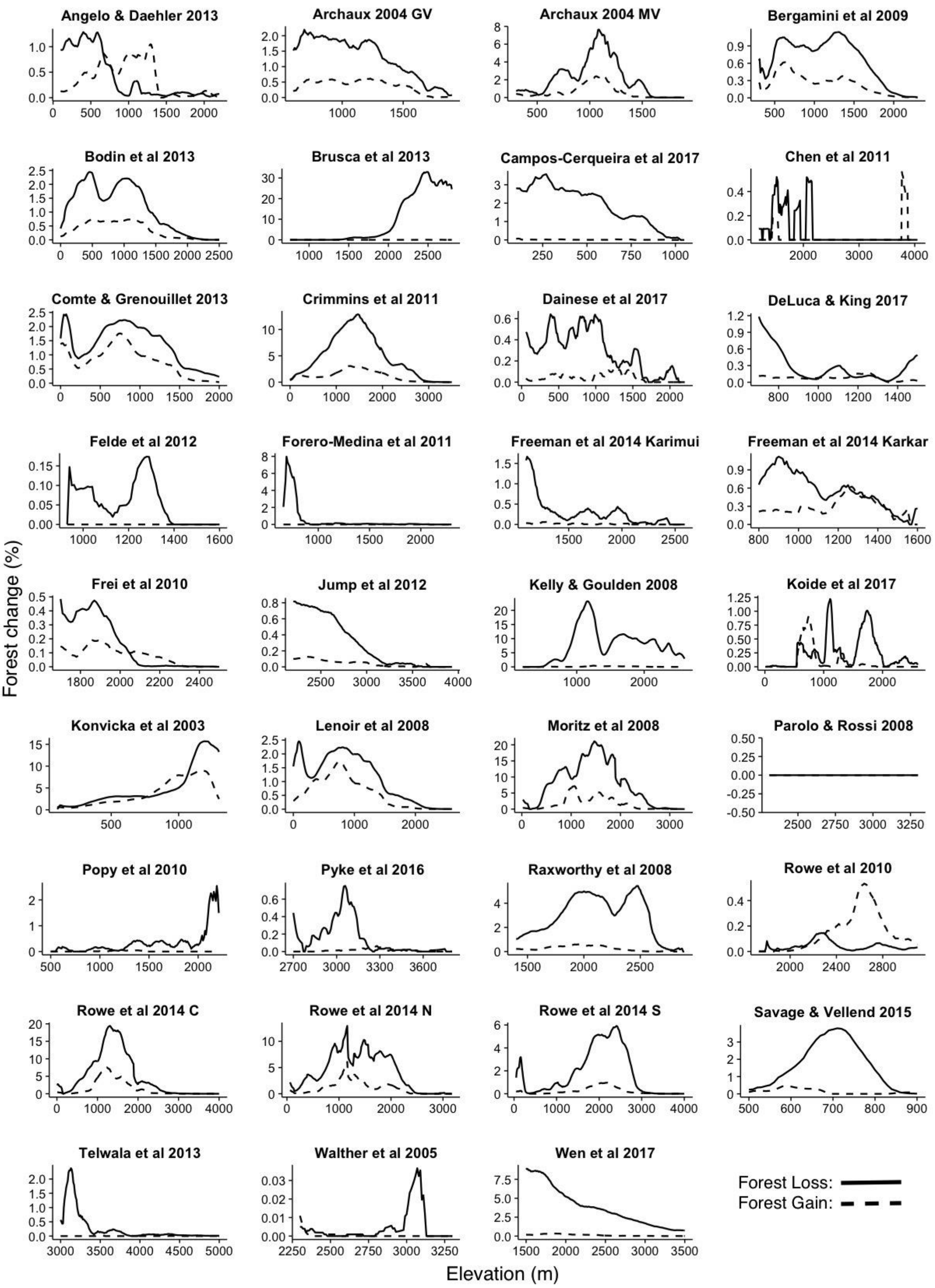


***Supplementary Figure 5. Forest cover change (gain and loss in %) profiles across elevation (10-m band) for the 35 study sites with species-level shift data.*** *Data plotted within the studied elevation range.*

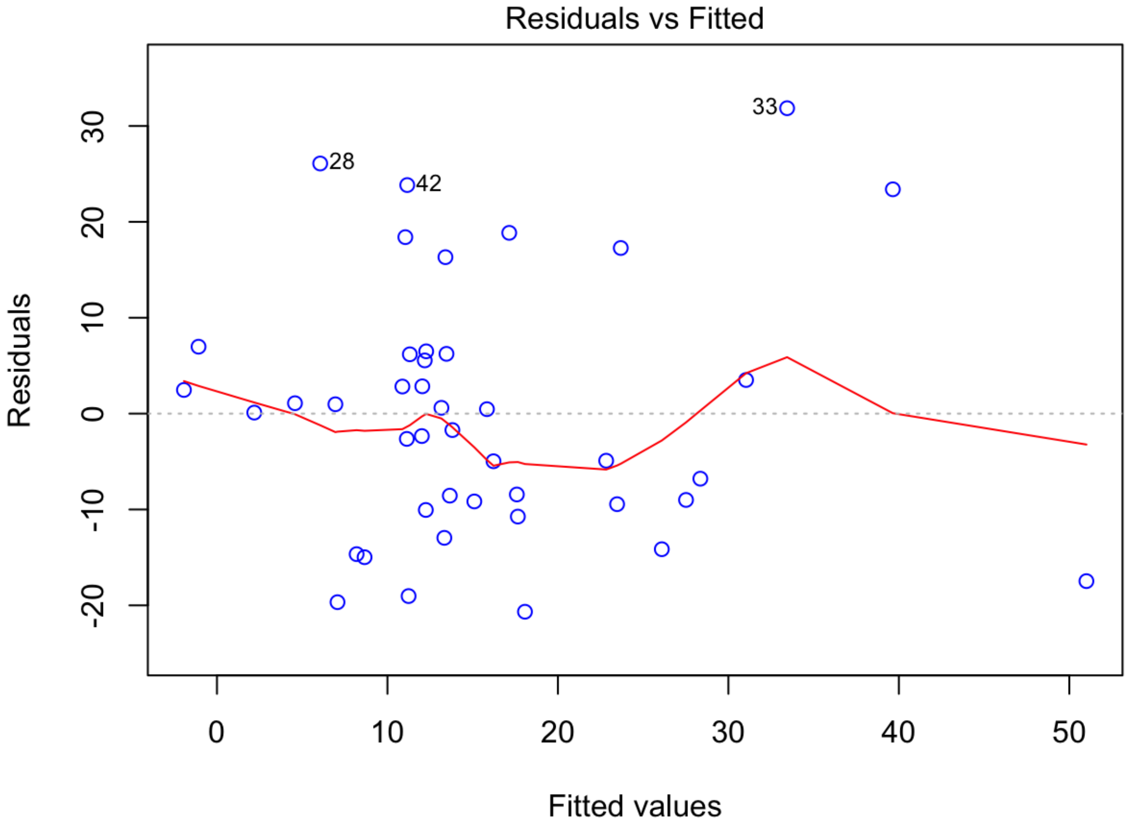


***Supplementary Figure 6. Diagnostic plot of residuals vs fitted values of the best model (model 2 in Table 1 and 2) in site-level analysis (n = 43).*** *Residuals equally distributed around the horizontal line.*

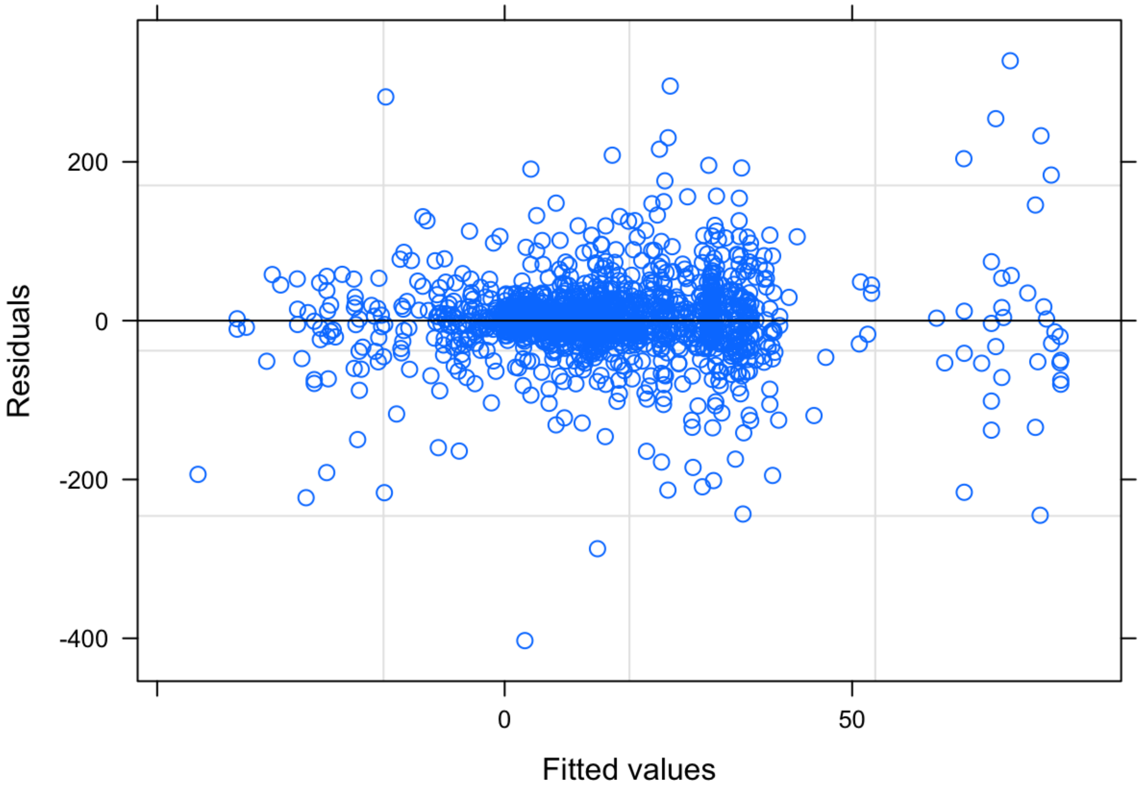


***Supplementary Figure 7. Diagnostic plot of residuals vs fitted values of the top model (model 1 in Supplementary Table 3) in species-level analysis (n = 2798).*** *Residuals equally distributed around the horizontal line.*

## Supplementary Tables

***Supplementary Table 1. Details of Model 2 in Table 1 (n = 43) (unscaled variables)*** *(ordinary least-square (OLS) regression model:* $F_{4,38} = 5.76$, $R^2 = 0.31$, $P = 0.001$)**.**

| Parameter | Estimate | Std. Error | t value | Pr (>\|t\|) |
|---|---|---|---|---|
| Intercept | 11.59 | 4.56 | 2.54 | 0.015 |
| Loss | -15.71 | 4.33 | -3.63 | <0.001 |
| Cover | 0.19 | 0.09 | 2.19 | 0.03 |
| T | -0.23 | 0.50 | -0.45 | 0.65 |
| Loss×T | 1.23 | 0.36 | 3.42 | 0.002 |

CCR: climate change rate, T: baseline temperature, Gain: forest gain percentage, Loss: forest loss percentage, Cover: forest cover percentage

***Supplementary Table 2. Details of Model 2 in Table 1 (n = 43) with sites weighted by the number of species included.*** *Predictor variables are scaled (cf. the scale() function in R) for comparison purposes (weighted ordinary least-square (OLS) regression model:* $F_{4,38} = 4.88$, $R^2 = 0.27$, $P = 0.003$*).*

| Parameter | Estimate | Std. Error | t value | Pr (>\|t\|) |
|---|---|---|---|---|
| Intercept | 16.24 | 3.02 | 5.37 | <0.001 |
| scale(Loss) | -12.71 | 4.01 | -3.17 | 0.003 |
| scale(Cover) | 5.71 | 3.94 | 1.45 | 0.16 |
| scale(T) | 14.87 | 5.33 | 2.79 | 0.008 |
| scale(Loss)×scale(T) | 16.88 | 7.20 | 2.34 | 0.02 |

T: baseline temperature, Loss: forest loss percentage, Cover: forest cover percentage.

*Supplementary Table 3. Linear mixed-effect models (LMMs) (fixed effect components) ranked by AICc values (ΔAICc<2), with the same random structure included in all models (1+T+Cover/Site).*

| Model | Parameters | $AIC_c$ | $\Delta AIC_c$ | Weight | $R^2_{marginal}$ | $R^2_{conditional}$ |
|---|---|---|---|---|---|---|
| 1 | Ref, Cover, Sdist, T | 28438.84 | | 0.14 | 0.05 | 0.23 |
| 2 | Ref, CCR, Cover, Sdist, T, CCR×Cover | 28439.24 | 0.41 | 0.12 | 0.05 | 0.24 |
| 3 | Ref, Cover, Loss, Sdist, T | 28439.83 | 0.99 | 0.09 | 0.05 | 0.24 |
| 4 | Cover, Sdist, T | 28439.89 | 1.05 | 0.08 | 0.05 | 0.23 |
| 5 | Ref, CCR, Cover, Sdist, T, CCR×Cover, CCR×T | 28440.02 | 1.18 | 0.08 | 0.06 | 0.24 |
| 6 | Ref, Cover, Loss, Sdist, T, Cover×Loss | 28440.28 | 1.44 | 0.07 | 0.05 | 0.24 |
| 7 | Ref, CCR, Cover, Sdist, T | 28440.34 | 1.51 | 0.07 | 0.05 | 0.23 |
| 8 | Ref, CCR, Cover, Loss, Sdist, T, CCR×Loss | 28440.54 | 1.70 | 0.06 | 0.05 | 0.23 |
| 9 | CCR, Cover, Sdist, T, CCR×Cover | 28440.58 | 1.75 | 0.06 | 0.05 | 0.25 |
| 10 | Ref, Loss, Sdist, T | 28440.59 | 1.75 | 0.06 | 0.03 | 0.24 |
| 11 | Ref, CCR, Cover, Loss, Sdist, T, CCR×Cover, CCR×Loss | 28440.67 | 1.83 | 0.06 | 0.05 | 0.23 |
| 12 | Ref, CCR, Cover, Loss, Sdist, T, CCR×Cover | 28440.68 | 1.85 | 0.06 | 0.05 | 0.24 |
| 13 | Ref, Cover, Sdist, T, Type | 28440.77 | 1.93 | 0.05 | 0.05 | 0.23 |

CCR: climate change rate, T: baseline temperature, Loss: forest loss percentage, Cover: forest cover percentage, Sdist: elevational distance to mountain summit, Type: taxa type (animal or plant), Ref: reference point (margin or centre).

*Supplementary Table 4. **Correlation matrix for the site-level analysis (n = 43)**, showing Pearson correlation coefficients, and the associated significance level, among numeric variables.*

| Variables | Shift.rate | CCR | Cover | Loss | Gain | T |
|---|---|---|---|---|---|---|
| Shift.rate | - | | | | | |
| CCR | -0.13 | - | | | | |
| Cover | 0.33* | 0.03 | - | | | |
| Loss | -0.08 | -0.28 | -0.04 | - | | |
| Gain | -0.15 | -0.06 | -0.10 | 0.56*** | - | |
| T | 0.36* | -0.04 | 0.49*** | 0.3 | 0.13 | - |

Shift.rate: response shift rate per decade, CCR: climate change rate, Cover: forest cover percentage, Loss: forest loss percentage, Gain: forest gain percentage, T: baseline temperature.
*p < .05. **p < .01. ***p < .001.

***Supplementary Table 5. Correlation matrix for the species-level analysis (n = 2,798)****, showing Pearson correlation coefficients, and the associated significance level, among numeric variables.*

| Variables | Shift.rate | CCR | Cover | Loss | Gain | T | Sdist |
|---|---|---|---|---|---|---|---|
| Shift.rate | - | | | | | | |
| CCR | -0.03 | - | | | | | |
| Cover | 0.03 | 0.06** | - | | | | |
| Loss | 0.02 | -0.11*** | 0.10*** | - | | | |
| Gain | 0.03 | 0.01 | 0.02 | 0.66*** | - | | |
| T | 0.08*** | -0.03 | 0.45*** | 0.19*** | 0.12*** | - | |
| Sdist | 0.10*** | 0.12*** | -0.28*** | 0.22*** | 0.44*** | 0.19*** | - |

Shift.rate: response shift rate per decade, CCR: climate change rate, Cover: forest cover percentage, Loss: forest loss percentage, Gain: forest gain percentage, T: baseline temperature, Sdist: elevational distance to mountain summit.

*p < .05. **p < .01. ***p < .001.

***Supplementary Table 6. Ordinary least-square (OLS) regression model outcomes restricted to forest ecosystems only (n = 29).*** *We applied the same stepwise model selection process as with the full dataset, and selected the best model fitting current dataset by AICc and* $R^2$*= 0.40.*

| Parameter | Estimate | Std. Error | t value | Pr (>\|t\|) |
|---|---|---|---|---|
| Intercept | 16.43 | 2.72 | 6.04 | <0.001 |
| scale(Loss) | -1.41 | 2.94 | -0.48 | 0.64 |
| scale(Cover) | 6.03 | 3.13 | 1.93 | 0.07 |
| scale(T) | 13.07 | 3.79 | 3.45 | 0.002 |
| scale(Loss)×scale(T) | 15.72 | 5.88 | 2.67 | 0.01 |

T: baseline temperature, Loss: forest loss percentage, Cover: forest cover percentage.

**Supplementary References**


1. Angelo, C. L. and Daehler, C. C. (2013). Upward expansion of fire-adapted grasses along a warming tropical elevation gradient. *Ecography* ***36***, 551–559.
2. Archaux, F. (2004). Breeding upwards when climate is becoming warmer: no bird response in the French Alps. *Ibis*, ***146***, 138–144.
3. Bergamini, A. et al. (2009). An elevational shift of cryophilous bryophytes in the last century – an effect of climate warming? *Divers. Distrib.* ***15***, 871–879.
4. Bodin, J. et al. (2013). Shifts of forest species along an elevational gradient in Southeast France: climate change or stand maturation? *J. Veg. Sci.* ***24***, 269–283.
5. Brusca, R. C. et al. (2013). Dramatic response to climate change in the Southwest: Robert Whittaker's 1963 Arizona Mountain plant transect revisited. *Ecol. Evol.* ***3***, 3307– 3319.
6. Campos-Cerqueira, M. et al. (2017). Have bird distributions shifted along an elevational gradient on a tropical mountain? *Ecol. Evol.* ***7***, 9914 - 9924.
7. Chen, I.-C. et al. (2011). Asymmetric boundary shifts of tropical montane Lepidoptera over four decades of climate warming. *Glob. Ecol. Biogeogr.* ***20***, 34–45.
8. Comte, L. and Grenouillet, G. (2013). Do stream fish track climate change? Assessing distribution shifts in recent decades. *Ecography* ***36***, 1236–1246.
9. Crimmins, S. M. et al. (2011). Changes in climatic water balance drive downhill shifts in plant species' optimum elevations. *Science* ***331***, 324–327.
10. Dainese et al. (2017) Human disturbance and upward expansion of plants in a warming climate. *Nat. Clim. Change* ***7***, 577–580
11. DeLuca, W. V., & King, D. I. (2017). Montane birds shift downslope despite recent warming in the northern Appalachian Mountains. *J. Ornitho* ***158***, 493-505.
12. Felde, V. A. et al. (2012). Upward shift in elevational plant species ranges in Sikkilsdalen, central Norway. *Ecography* ***35***, 922–932.
13. Forero-Medina, G. et al. (2011). Elevational ranges of birds on a tropical montane gradient lag behind warming temperatures. *PloS ONE* ***6***, e28535.
14. Freeman, B. G., & Freeman, A. M. C. (2014). Rapid upslope shifts in New Guinean birds illustrate strong distributional responses of tropical montane species to global warming. *Proc. Natl. Acad. Sci.* ***111***, 4490-4494.
15. Frei, E. et al. (2010). Plant species' range shifts in mountainous areas: all uphill from here? *Bot. Helvetica* ***120***, 117–128.
16. Holzinger, B. et al. (2008). Changes in plant species richness over the last century in the eastern Swiss Alps: elevational gradient, bedrock effects and migration rates. *Plant Ecol.* ***195***, 179–196.
17. Jump, A. S. et al. (2012). Rapid altitudinal migration of mountain plants in Taiwan and its implications for high altitude biodiversity. *Ecography* ***35***, 204–210.
18. Kelly, A. E. and Goulden, M. L. (2008). Rapid shifts in plant distribution with recent climate change. *Proc. Natl. Acad. Sci.* ***105***, 11823–11826.
19. Koide, D. et al. (2017). An upward elevational shift of native and non-native vascular plants over 40 years on the island of Hawai'i. *J. Veg. Sci.* ***28***, 939 – 950.
20. Konvicka, M. et al. (2003). Uphill shifts in distribution of butterflies in the Czech

Republic: effects of changing climate detected on a regional scale. *Glob. Ecol. Biogeogr.* ***12***, 403–410.
21. Lenoir, J. et al. (2008). A significant upward shift in plant species optimum elevation during the 20th century. *Science* ***320***, 1768–1771.
22. Maggini, R. et al. (2011). Are Swiss birds tracking climate change? Detecting elevational shifts using response curve shapes. *Ecol. Model.* ***222***, 21–32.
23. Massimino, D. et al. (2015). The geographical range of British birds expands during 15 years of warming. *Bird Stud*, ***62***, 523-534.
24. Molina‐Martinez, A. et al. (2016). Changes in butterfly distributions and species assemblages on a Neotropical mountain range in response to global warming and anthropogenic land use. *Divers. Distributions* ***22***, 1085-1098.
25. Moritz, C. et al. (2008). Impact of a century of climate change on small-mammal communities in Yosemite National Park, USA. *Science* ***322***, 261–264.
26. Morueta-Holme et al. (2015) Strong upslope shifts in Chimborazo's vegetation over two centuries since Humboldt. *Proc. Natl. Acad. Sci.* ***112***, 12741–12745.
27. Parolo, G. and Rossi, G. (2008). Upward migration of vascular plants following a climate warming trend in the Alps. *Basic Appl. Ecol.* ***9***, 100–107.
28. Ploquin, E. F. et al. (2013). Bumblebee community homogenization after uphill shifts in montane areas of northern Spain. *Oecologia* ***173***, 1649–1660.
29. Popy, S. et al. (2010). A weak upward elevational shift in the distributions of breeding birds in the Italian Alps. *J. Biogeogr.* ***37***, 57–67.
30. Pyke, G. H. et al. (2016). Effects of climate change on phenologies and distributions of bumble bees and the plants they visit. *Ecosphere* ***7***, e01267.
31. Raxworthy, C. J. et al. (2008). Extinction vulnerability of tropical montane endemism from warming and upslope displacement: a preliminary appraisal for the highest massif in Madagascar. *Glob. Change Biol.* ***14***, 1703–1720.
32. Rowe, R. J. et al. (2010). Range dynamics of small mammals along an elevational gradient over an 80-year interval. *Glob. Change Biol.* ***16***, 2930–2943.
33. Rowe, K. C. et al. (2015). Spatially heterogeneous impact of climate change on small mammals of montane California. *Proc. R. Soc. B* ***282***, 20141857.
34. Savage, J. & Vellend, M. (2015). Elevational shifts, biotic homogenization and time lags in vegetation change during 40 years of climate warming. *Ecography* ***38***, 546-555.
35. Telwala, Y. et al. (2013). Climate-induced elevational range shifts and increase in plant species richness in a Himalayan biodiversity epicentre. *PLoS ONE* ***8***, e57103.
36. Walther, G.-R. et al. (2005). Trends in the upward shift of alpine plants. *J. Veg. Sci.* ***16***, 541– 548.
37. Wen, Z. et al. (2017). Heterogeneous distributional responses to climate warming: evidence from rodents along a subtropical elevational gradient. *BMC Ecol.* ***17***, 17.
38. Wilson, R. J. et al. (2005). Changes to the elevational limits and extent of species ranges associated with climate change. *Ecol. Lett.* ***8***, 1138–1146.
39. Zuckerberg, B. et al. (2009). Poleward shifts in breeding bird distributions in New York State. *Glob. Change Biol.* ***15***, 1866–1883.
40. Elsen, P. R. & Tingley, M. W. (2015). Global mountain topography and the fate of

montane species under climate change. *Nat. Clim. Change* ***5***, 772-776.

41. Hansen, M. C., et al. (2013). High-resolution global maps of 21st-century forest cover change. *Science* ***342,*** 850-853.
42. Hijmans, R. J. et al. (2005). Very high resolution interpolated climate surfaces for global land areas. *Int. J. Climatol.* ***25****,*1965-1978.
43. R Core Team (2016). R: A language and environment for statistical computing. R Foundation for Statistical Computing, Vienna, Austria. URL: https://www.R-project.org/.
