# Supplementary material for: Land-use change interacts with climate to determine elevational species redistribution
Source: Nat Commun. 2018 Apr 3;9:1315. doi: 10.1038/s41467-018-03786-9 (PMC5883048; doi:10.1038/s41467-018-03786-9)
Supplement: Supplementary file 3 — Description of Additional Supplementary Files(PDF 171 kb) [file 41467_2018_3786_MOESM3_ESM.pdf]

Description of Additional Supplementary Files

Supplementary Software. R script for running the analyses.

Supplementary Data 1. The data used for site-level analysis; available on Dryad [doi:10.5061/dryad.k8g2672].

Supplementary Data 2. The data used for species-level analysis; available on Dryad [doi:10.5061/dryad.k8g2672].
